# Effectiveness of endothelial progenitor cell culture under microgravity for improved angiogenic potential

**DOI:** 10.1038/s41598-018-32073-2

**Published:** 2018-09-24

**Authors:** Hiroko Hagiwara, Akira Higashibata, Shiho Ogawa, Shigeyuki Kanazawa, Hiroshi Mizuno, Rica Tanaka

**Affiliations:** 1 Department of Plastic and Reconstructive Surgery, School of Medicine, Juntendo University, Tokyo, Japan; 2 Center for Genomic and Regenerative Medicine, School of Medicine, Juntendo University, Tokyo, Japan; 3 JEM Utilization Center, Human Spaceflight Technology Directorate, Japan Aerospace Exploration Agency, Ibaraki, Japan

## Abstract

Endothelial progenitor cell (EPC) transplantation is beneficial for ischemic diseases such as critical limb ischemia and ischemic heart disease. The scarcity of functional EPCs in adults is a limiting factor for EPC transplantation therapy. The quality and quantity culture (QQc) system is an effective *ex vivo* method for enhancing the number and angiogenic potential of EPCs. Further, microgravity environments have been shown to enhance the functional potential of stem cells. We therefore hypothesized that cells cultured with QQc under microgravity may have enhanced functionality. We cultured human peripheral blood mononuclear cells using QQc under normal (E), microgravity (MG), or microgravity followed by normal (ME) conditions and found that ME resulted in the most significant increase in CD34+ and double positive Dil-Ac-LDL-FITC-Ulex-Lectin cells, both EPC markers. Furthermore, angiogenic potential was determined by an EPC-colony forming assay. While numbers of primitive EPC-colony forming units (pEPC-CFU) did not change, numbers of definitive EPC-CFU colonies increased most under ME conditions. Gene-expression profiling also identified increases in angiogenic factors, including vascular endothelial growth factor, under MG and ME conditions. Thus, QQc along with ME conditions could be an efficient system for significantly enhancing the number and angiogenic potential of EPCs.

## Introduction

Endothelial progenitor cells (EPCs) are responsible for vasculogenesis in embryos and adults. New drug and angioplasty therapies use EPC cell transplantation in angiogenic therapy^1–9^. EPC transplantation is performed as neovascularization therapy for ischemic diseases such as critical limb ischemia and ischemic heart disease^2–4^.

Since there are few functional EPCs in adults, EPC transplantation therapy is limited. Further, aging, diabetes, hyperlipidemia, and cardiovascular disease all contribute to the declines in both the number and functionality of EPCs^10–12^. To overcome this problem, several conditions for the *ex vivo* cultivation and expansion of EPCs have been developed; however, these techniques yield insufficient cell numbers and angiogenic potential^13^.

In recent years, a quality and quantity culture (QQc) system, an *ex vivo* expansion culture method, has been used to increase the number of EPCs and improve their angiogenic potential^4,6,14^. This method involves culturing cells in a serum-free culture medium enriched with optimal cytokines and growth factors for 7 days, and requires only a small volume of peripheral blood for autologous therapy. Cultivating peripheral blood mononuclear cells (PBMNCs) using the QQc method has resulted in increased total EPC-colony forming units (tEPC-CFU) and a six-fold increase in the total angiogenic potential of the EPCs, compared to control cells^4^. The QQc system also resulted in an increase in the expression of genes that are involved in angiogenesis, such as vascular endothelial growth factor (*VEGF*), insulin-like growth factor 1 (*IGF-1*), and matrix metallopeptidase-9 (*MMP-9*)^4^. Other research involving cell transplantation in a femoral cartilage defect mouse model showed that cells that were expanded with QQc had increased potential for accelerating fracture healing^15^. These results suggest that the transplantation of cells that are expanded using the QQc method is more efficient than conventional cell transplantation methods. Nevertheless, the establishment of a more efficient cell cultivation method is required so that this technique can be developed for clinical applications.

Microgravity has been reported to improve the numbers of human hematopoietic progenitor cells^16^ and the functions of stem cells, such as mesenchymal stem cells (MSCs) and bone marrow stromal cells^17,18^. Human MSCs that are grown under a microgravity environment proliferate at a higher rate than those grown under normal gravity, maintain their differentiation potency when transplanted into the femoral cartilage defect mouse model, and are able to repair defects in the femoral cartilage^17^. The microgravity environment has also enhanced the neural repair potential of bone marrow stromal cells and increased their surface expression of C-X-C chemokine receptor type 4 (CXCR4), which is involved in cell migration^18^. Another study cultivated human umbilical vein endothelial cells (HUVEC) under a microgravity environment and found accelerated neovascularization that resulted from increased lumen-formation^19^. Based on these findings, we hypothesized that the QQc method may be more effective for EPC expansion *ex vivo* under microgravity conditions, compared to normal gravity conditions. Our study is the first to show that the QQc method combined with microgravity conditions is a superior method for EPC expansion.

## Results

### Effects of QQc and microgravity on total cell numbers

**Figure 1:**
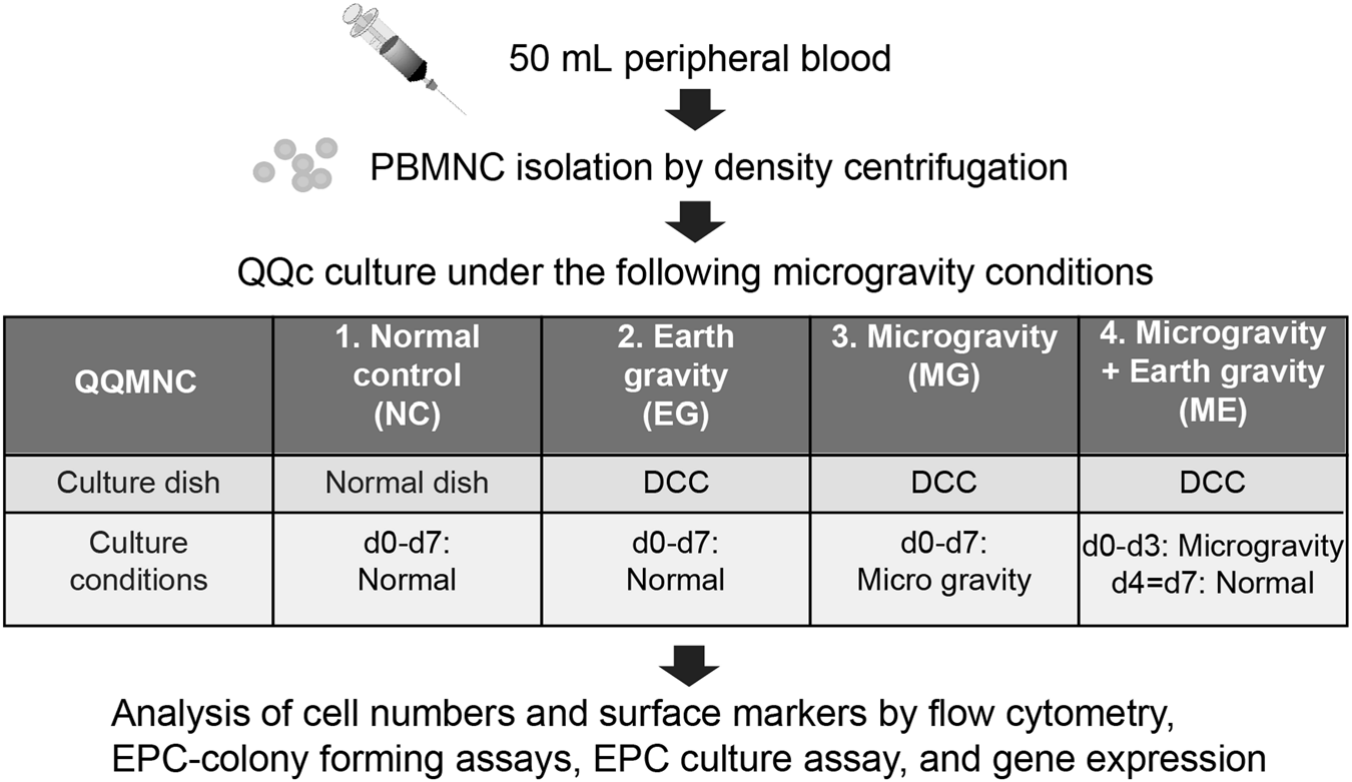
The schematic of the culture protocol under microgravity and earth (normal) gravity. Abbreviations: PBMNC = peripheral blood mononuclear cells; QQMNC = mononuclear cells cultured under quality and quantity culture conditions; VEGF = endothelial growth factor; TPO = thrombopoietin; SCF = stem cell factor; IL-6 = interleukin-6; DCC = disposable cultivation chamber.

**Figure 2:**
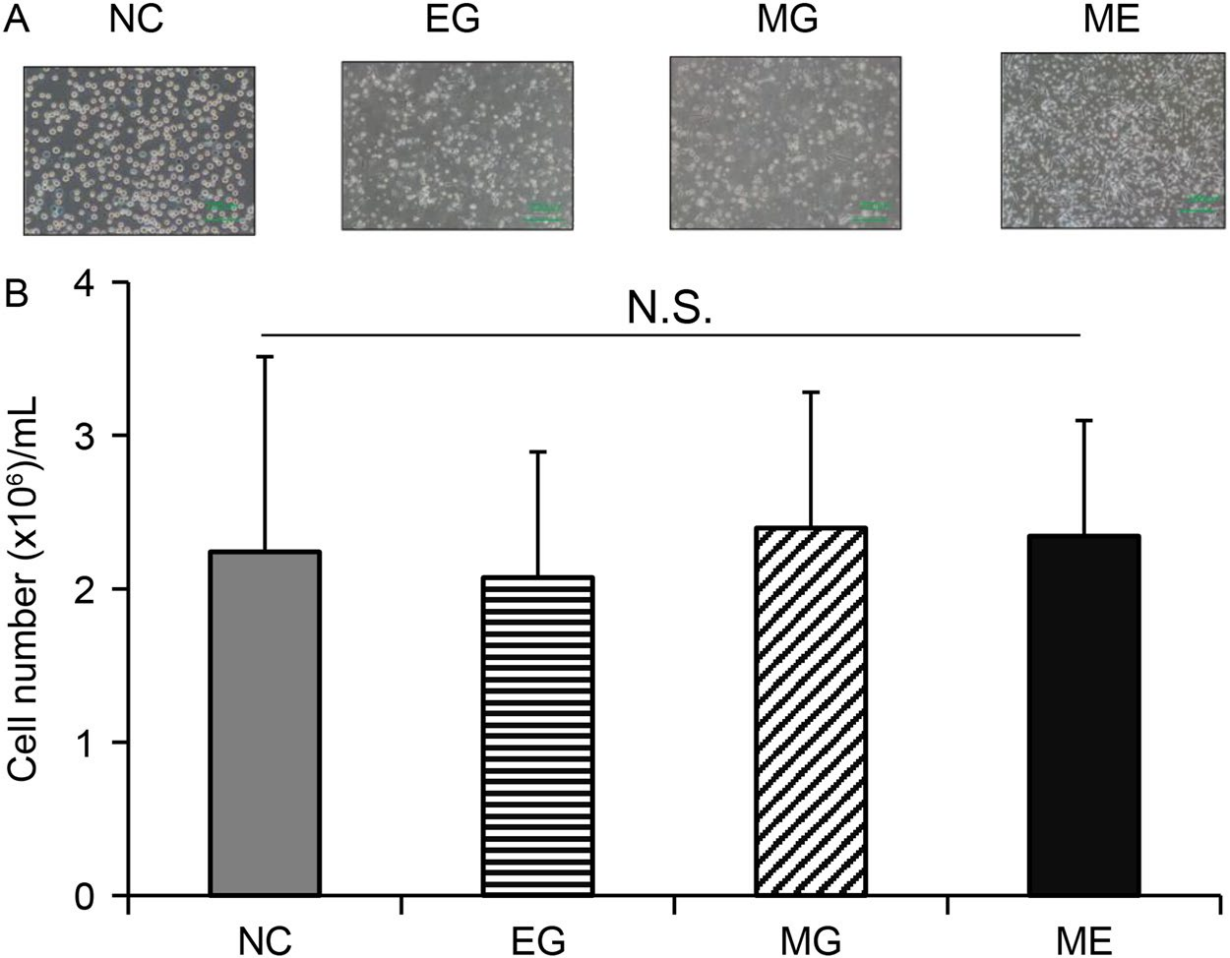
Total cell numbers of PBMNCs and QQMNCs cultured under different gravity conditions. There were no significant differences in the total cell number after seven days of QQc. (**A**) Representative images of the cultures at 100x magnification. (**B**) Total cell counts after culture. Values are the means ± SD from seven samples. Data shown is representative of three independent experiments. Abbreviation: EG = earth (normal) gravity; ME = microgravity and normal gravity; MG = microgravity.

A 3D–Clinostat, which is a multidirectional G force generator, was used to simulate microgravity conditions. As shown in Fig. 1, cells were cultured under four different conditions: NC – normal control, EG – earth gravity, MG – Microgravity, and ME – microgravity and earth gravity. There were no significant differences in total cell numbers after seven days of QQc. Cell growth was similar among all four groups, with 2.24 ± 0.202 × 10^6^/mL cells in the control group, 2.08 ± 0.26 in the EG group, 2.35 ± 0.28 in the MG group, and 2.40 ± 0.24 in the ME group (Fig. 2A,B).

### Microgravity stimulation increases the number of CD34+ cells

**Figure 3:**
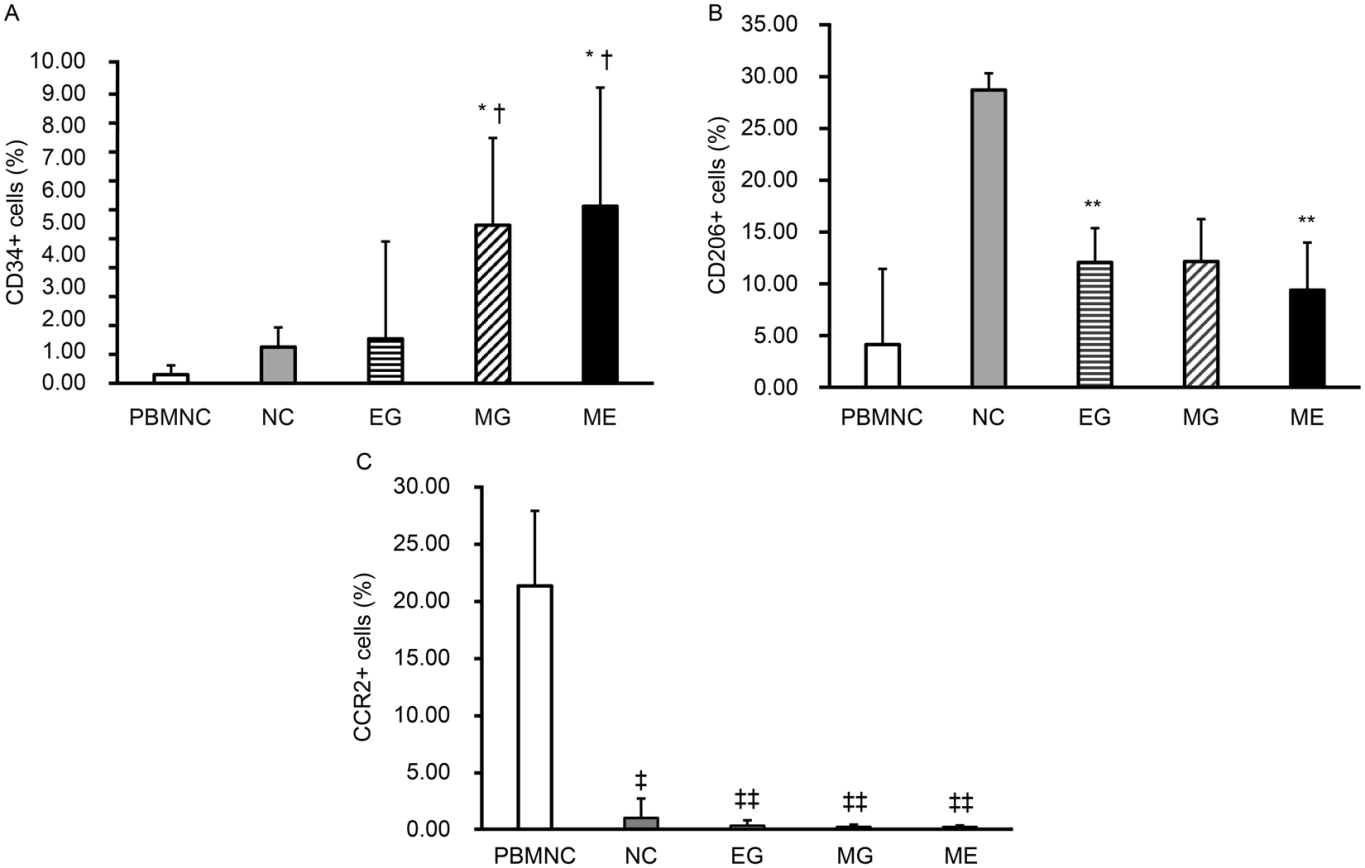
Flow cytometry analysis of PBMNCs and QQMNCs cultured under different gravity conditions. (**A**) Expression of CD34+ cells (%). Expression of CD34 significantly increased in the cells of the MG and ME groups compared with those of the PBMNC and EG groups. *p < 0.05 versus control group. ^†^p < 0.05 versus EG group. (**B**) Expression of CD206+ cells (%). Expression of CD206 was lower in the cells of the MG group compared to that of the control. **p < 0.01 versus control group. (**C**) Expression of CCR2+ cells (%). CCR2 expression significantly decreased in the cells of the EG, MG, and ME groups compared to those of the PBMNC group. ^‡^p < 0.05, ^‡‡^p < 0.01 versus PBMNC. All surface marker values are the means ± SD from five samples. Data shown is representative of three independent experiments.

There was a significant increase (p < 0.01) in the percentage of CD34+ cells, an EPC marker, in the MG group (4.90 ± 2.71) compared with the control (1.12 ± 0.61) group (Fig. 3A). The percentages were significantly different (p < 0.05) when the ME group (5.50 ± 3.68) was compared to the control (1.12 ± 0.61) group. Significant differences (p < 0.05) were also observed between the EG (1.38 ± 3.02) and MG (4.90 ± 2.71) groups and between the EG (1.38 ± 3.02) and ME (5.50 ± 3.68) groups (Fig. 3A).

There were also differences in the percentages of CD206+ cells, an anti-inflammatory macrophage marker, among all groups (Fig. 3B). Compared with the PBMNC group (4.12 ± 7.28), the percentage was higher in the control (28.62 ± 1.61; p < 0.005), EG (12.02 ± 3.30; p < 0.005), MG (12.11 ± 4.08; p < 0.005), and ME (9.35 ± 4.58, p < 0.005) groups (Fig. 3B). On the other hand, compared to that of the control (28.62 ± 1.61) group, the percentage of CD206+ was lower in the EG (12.02 ± 3.30; p < 0.005), MG (12.11 ± 4.08; p < 0.005), and ME (9.35 ± 4.58, p < 0.005) groups (Fig. 3B).

It has been previously reported that the percentage of the inflammatory macrophage marker CCR2 decreases in cells cultured with the QQc system^4^. Compared with PBMNC (21.28 ± 9.54), we also observed that the percentage of CCR2 was lower in the control (0.98 ± 0.83; p < 0.05), EG (0.30 ± 0.39; p < 0.01), MG (0.20 ± 0.27; p < 0.01), and ME 0.19 ± 0.29; p < 0.01) groups (Fig. 3C).

Further, compared to the control (0.98 ± 0.83) group, the greatest decrease in the percentage of CCR2 + cells occurred in the MG (0.20 ± 0.27; p = 0.051) and ME (0.19 ± 0.29; p = 0.052) groups, while the number of cells in the EG (0.30 ± 0.39; p = 0.063) group also decreased (Fig. 3C).

CD31 is a known vascular marker. We observed a significant decrease in the percentage of CD31 + cells in the ME group compared to those of the control group (56.92 ± 8.04 vs 68.40 ± 5.07; p < 0.05); however, no differences were seen in the percentages of CXCR4+ cells (involved in cell migration) or CD3+ cells (Supplementary Table S1).

### Microgravity stimulation increases the vasculogenic potential of QQMNCs

**Figure 4:**
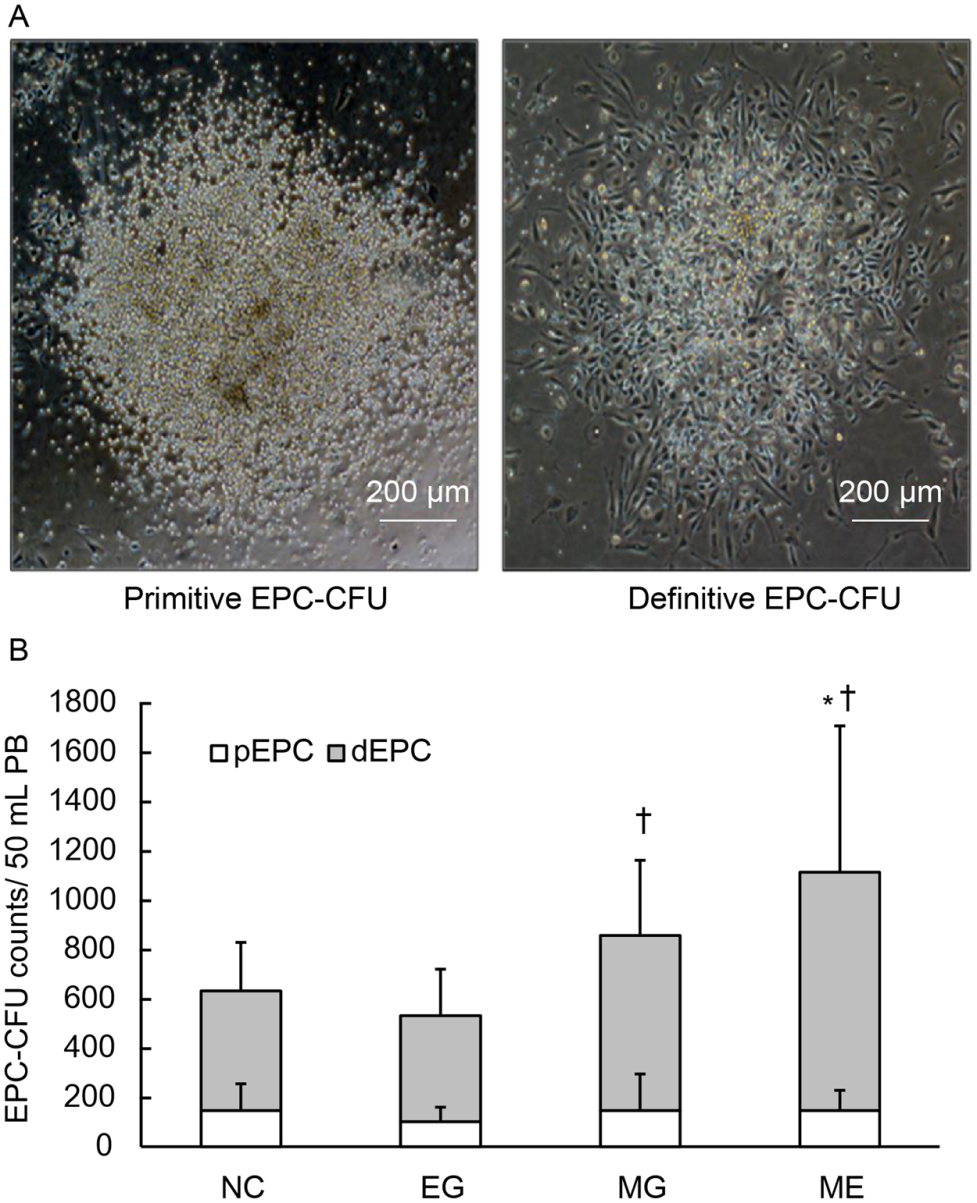
EPC-colony forming assay. (**A**) Representative images of pEPC-CFU and dEPC-CFU at 100x magnification. pEPC-CFU = primitive endothelial progenitor cell colony forming unit; dEPC-CFU = definitive endothelial progenitor cell colony forming unit. (**B**) pEPC-CFU and dEPC-CFU counts generated from QQMNCs in 100 mL peripheral blood. (**C**) Total EPC-CFU counts generated from QQMNCs in 100 mL peripheral blood. *p < 0.05 versus normal control group. ^†^p < 0.05 versus EG group. Values are the means ± SD from six samples. Data shown is representative of three independent experiments.

Compared to PBMNCs, QQMNCs (cells cultured under QQc conditions) have a higher potential for EPC-CFU, which represents the angiogenic potential of EPCs^4^. EPC-colony forming assay (EPC-CFA) were used to investigate whether changes would occur in the EPC-CFU counts of QQMNC when they were cultured under different environments. Two types of colonies, primitive-EPC (pEPC)-CFU and definitive-EPC (dEPC)-CFU, were generated with EPC-CFA. Each of these types had its own unique cell morphology and function (Fig. 4A).

Of great interest were the results for the ME group. This group underwent three days of QQc under microgravity and then four days under earth (normal) gravity. The ME group had a significantly higher number of dEPC-CFU (967.1 ± 593.5) compared to the control (484.3 ± 197.0; p < 0.05) group (Fig. 4B). This was also evident when the ME group was compared to the EG (429.8 ± 189.0; p < 0.01**)** group (Fig. 4B). The dEPC-CFU numbers in the MG group (711.0 ± 304.7) were also higher than those of the control (484.3 ± 197.0) and EG (429.8 ± 189.0**)** groups, but these results were not statistically significant (Fig. 4B).

No significant differences were found among any of the QQMNC groups when the pEPC results were examined (Fig. 4B).

The total EPC-CFU numbers were significantly higher in the MG (857.5 ± 381.9; p < 0.05) and ME (1,113.5 ± 581.4; p < 0.01) groups than in the control (631.7 ± 410.1) group. These numbers were lower in the EG (531.6 ± 224.5) group (Fig. 4C); however, this was not statistically significant. The total EPC-CFU in the MG group (857.5 ± 381.9) was significantly higher than in the EG (531.6 ± 224.5; p < 0.05) group (Fig. 4C). Taken together, our results demonstrate that total dEPC numbers were higher when cells were cultivated under a microgravity environment than under an earth (normal) environment.

### EPC increased under ME environments

**Figure 5:**
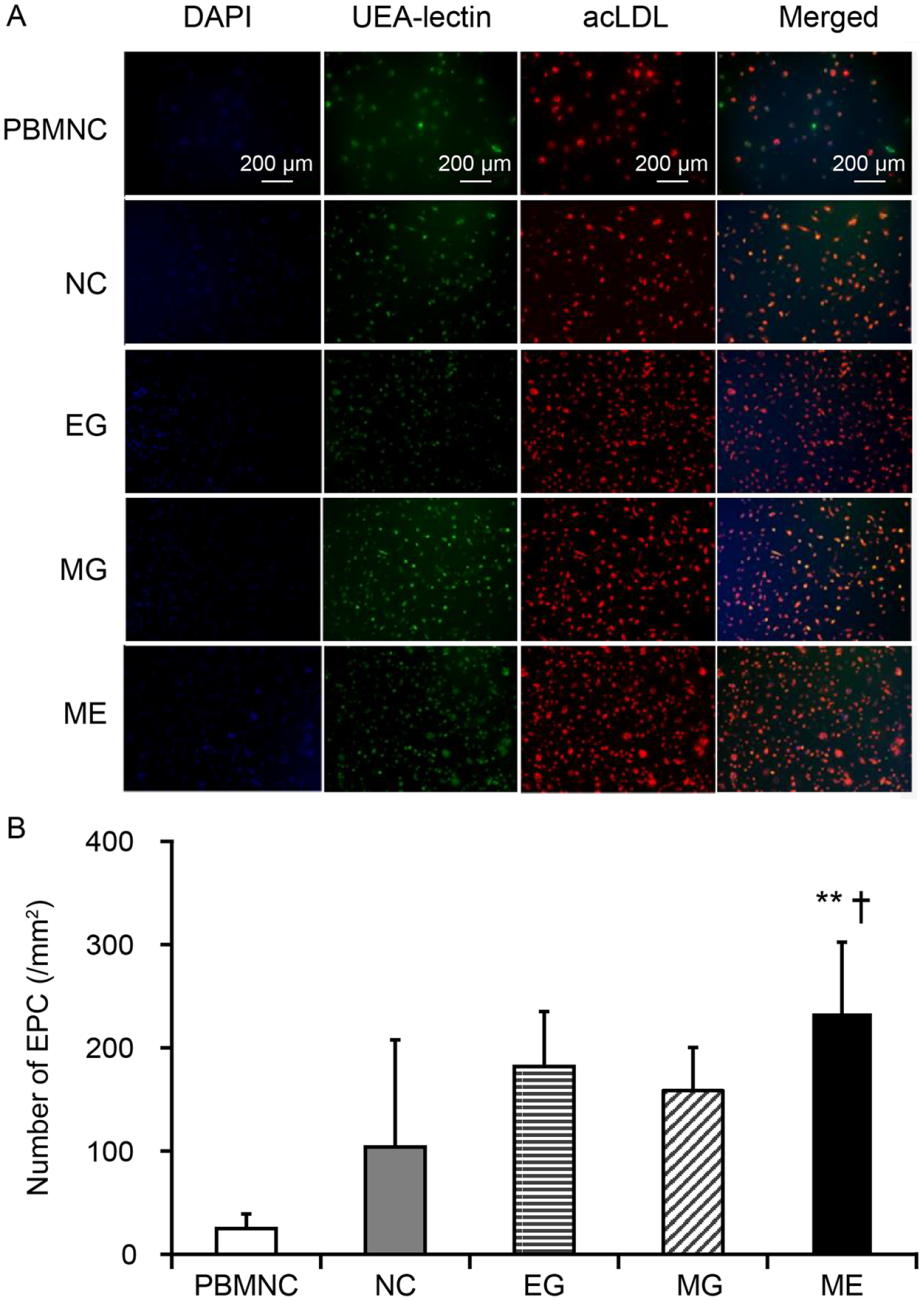
EPC-culture assay. (**A**) Representative images of PBMNC and QQMNC cultured using the EPC culture assay at 100x magnification. (**B**) The bar graph shows the number of EPCs in each group. Merged cells were counted as EPCs. **p < 0.01 versus normal control group. ^†^p < 0.05 versus EG group. Values are the means ± SD from five samples, four wells counted/sample.

A characteristic of EPC is that they take up Dil-ac-LDL and bind with FITC-Ulex-Lectin. We observed that compared to that of PBMNCs, there were significantly higher numbers of the double positive cells in QQMNCs (Fig. 5A). Our results demonstrate that the number of EPCs were higher when cultured under ME environments. The ME group (233.4 ± 68.9) had a significant higher number of double-positive cells compared to the control (104 ± 103.7; p < 0.01) and EG (182.1 ± 53.1; p < 0.05) groups (Fig. 5).

### Microgravity increases the expression of vasculogenic factors and suppresses inflammatory cytokines

Previous research has shown that angiogenic factors such as VEGF, IGF, and MMP-9 increase with the QQMNC method and that the expression of inflammatory factors, such as TGF-β, and TNF-α, declines^4^. Previous studies have also shown that in a microgravity environment, changes occur in the expression of a variety of genes, including *TNF-α* and *SOD-1*^20^. Our results show that a temporary microgravity stimulus increases the gene expression of vasculogenic factors and suppresses the expression of inflammatory cytokines.

**Figure 6:**
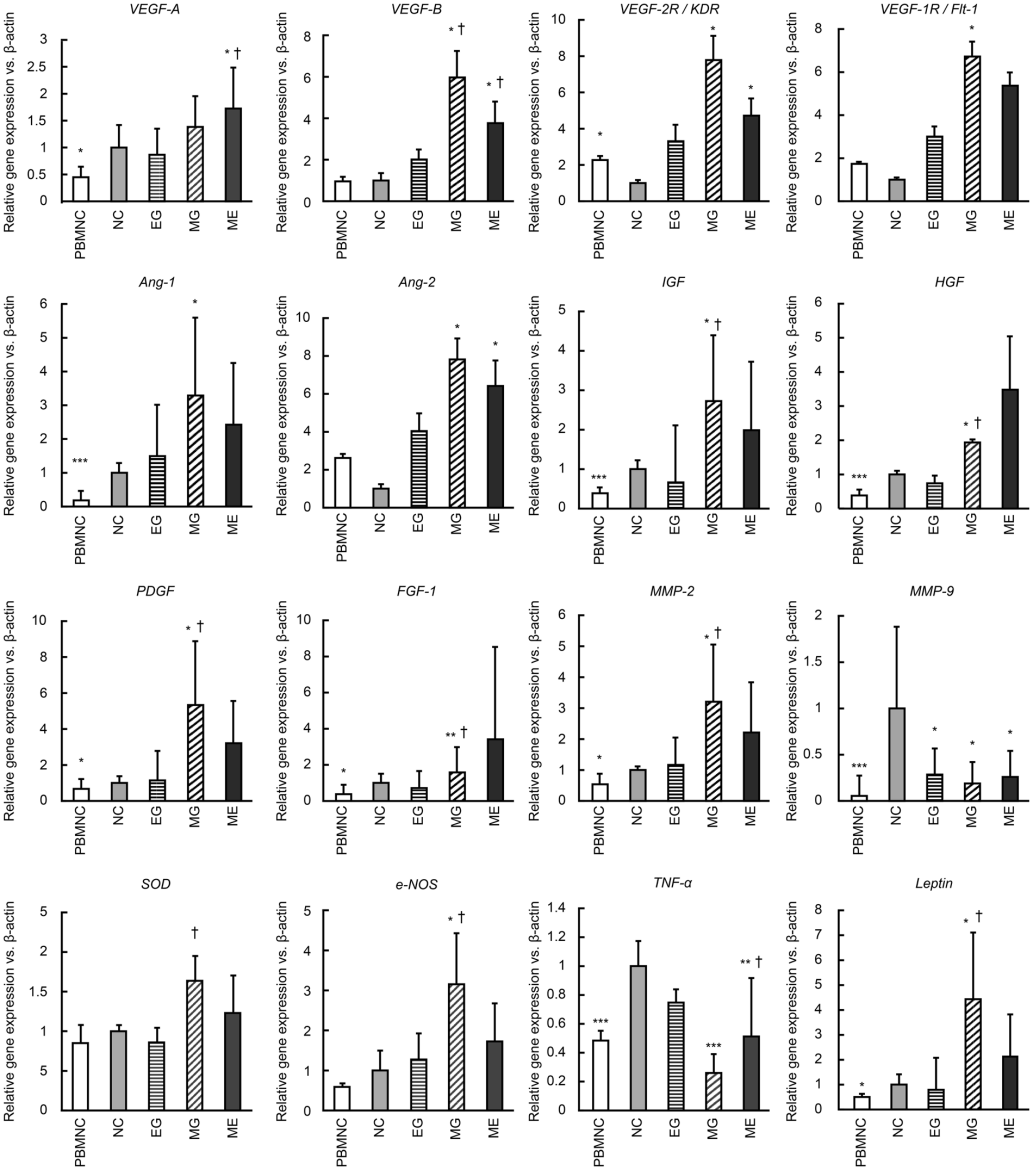
Quantitative PCR for PBMNC and QQMNC under different gravity conditions. *p < 0.05, **p < 0.01, ***p < 0.005 versus normal control group. ^†^p < 0.05 versus EG group. Values are the means ± SD from five samples. Data shown is representative of three independent experiments.

Compared to that in the control group, the expression of *VEGF-A, VEGF-2R/KDR, VEGF-1R/Flt-1, IGF, HGF, FGF-1, MMP-2, PDGF, e-NOS, Leptin, and SOD-1* were higher in the MG group (Fig. 6). Conversely, *TNF-α*, and inflammatory cytokines, were significantly lower in the ME group than in the control group (Fig. 6). The ME group also showed a higher expression of *VEGF-A* (1.72 ± 0.76) compared to the control group (Fig. 6). On the other hand, the control group showed higher expression of *MMP-9*, a factor involved in neovascularization and tissue repair (Fig. 6).

## Discussion

Our study is the first study to show that culturing EPC under QQc and microgravity conditions can enhance the number and vasculogenic potential of the EPCs.

Cell transplantation therapy using EPC is widely used as neovascularization therapy for a variety of ischemic diseases. Numerous clinical studies are currently underway involving the use of EPC, bone marrow mononuclear cells, PBMNC cultures, and CD34+ cells. Nevertheless, it is difficult to isolate sufficient EPCs for transplantation therapy^1–12^.

The QQc method is an *in vitro* vascular endothelial expansion culture method that aims to resolve the problems related to EPC transplantation^4^. The use of QQc results in improvements in the function and angiogenic potential of EPCs and increases the EPC expansion rate, which together suggest that QQc can be an effective method prior to EPC transplantation^4,6,14^.

Prior studies have shown that extended stays in outer space cause reductions in bone and muscle mass, anaemia, suppression of the immune system, and suppression in cell division and proliferation^21,22^. However, other studies have shown that microgravity simulation over a relatively short period of time accelerates cell expansion while maintaining the pluripotency of immature cells such as stem cells^17,18,23^. We therefore investigated whether the QQc method could be combined with microgravity environments to produce better results. Our results showed that PBMNCs cultured with the QQc method under a microgravity environment resulted in a significant increase in CD34+ cell numbers. These results are supported by data in the literature^17,23–25^ and showed that highly non-differentiated CD34+ cells undergo accelerated expansion upon microgravity simulation, while still maintaining their undifferentiated state. The data from our study showed that when cells were cultured in a normal environment after exposure to microgravity, there was a further improvement in the angiogenic potential of EPCs. There were higher numbers of CD34+ cells and significant increases in dEPC-CFU and tEPC-CFU. Since CD34+ cells are essential for EPC-CFUs, it is likely that the number of EPC-CFUs was dependent on the number of CD34+ cells, and the undifferentiated state was maintained under microgravity. However, other cells such as anti-inflammatory macrophages may also generate EPC-CFUs, and this aspect needs to be further studied. These results were similar to those reported in the literature, where cells under a normal gravity environment were observed to undergo differentiation^23^. In another study, HUVECs exposed to microgravity simulation had accelerated angiogenic potential^19^. This suggests that CD34+ cells with high non-differentiability can increase when exposed to microgravity. Our results show an additional improvement with exposure to microgravity followed by normal gravity, where the differentiation was further accelerated, which in turn raised the angiogenic potential of the EPCs.

While our results confirmed a reduction in *TNF-α* expression in the microgravity group, we did not find changes in the expression of transcription factors, such as *Oct4, Sox2, or NF-κB*. This may have occurred because unlike other studies where only one cell type was used, QQMNC populations contain many different cell types, and changes in the gene expression of specific cells may have been potentially masked. Future studies should investigate the gene expression changes in the PBMNCs that are cultured with QQc and remain undifferentiated under a microgravity environment. Furthermore, changes involved in the acceleration of the expansion potential of these cells should also be investigated. Studies have shown that the gene expression of angiogenic factors, including *VEGF*, angiopoietin, and matrix metalloproteinases (*MMP-2*, *MMP-9*), increases under microgravity environments^4,24–28^, while the expression of *TNF-α* and *TGF-β* decrease in this environment^20^. Our results were consistent with those reported in the literature. We found that cells that were cultured under microgravity conditions had increased expression of *VEGF, KDR, Flt-1*, angiopoietin, *HGF, FGF-1, MMP-2*, and *e-NOS*, and decreased expression of *TNF-α* and *TGF-β*. While *MMP-2* levels increased, *MMP-9* levels decreased. The decreases in *MMP-9* levels that we observed is in contrast to previous studies^29^, and it is likely that the differences in these results could be due to differences in the experimental setups or differences in the cell types that were studied.

There were a few limitations to our study. First, our experiments were carried out *in vitro*. To determine the true functionality of the QQMNC with microgravity, *in vivo* experiments need to be carried out. Second, we used a 3D-clinostat to simulate microgravity, whereas other studies used different types of equipment, including rotating wall vessels and parabolic flight campaign (PFC) equipment^17,18,23–26,30^, which could affect the results. Previous microgravity results have also been contradictory, and these reports have been extensively reviewed already^31,32^. Data from astronauts on space shuttles^30^ differ from the results that are obtained in some simulated microgravity environments. Thus, the technique that is used to create the microgravity conditions and the cell types that are studied could contribute to the differences in these results. Furthermore, while the QQc conditions have been previously optimized, the additional effect of microgravity may require further optimization of the culture conditions. It is likely that by changing the culture times or the concentrations of recombinant proteins in the culture media might result in more effective EPCs. It will therefore be important to understand the differences and determine the correct conditions for obtaining the maximum potential of these cells. Future studies could include examining other PBMNC cell types (apart from CD34+ cells) that may have angiogenic potential. In addition, studies could be carried out on other cells to determine if they exhibited EPC-like characteristics when cultured in microgravity conditions and subsequently cultured in earth (normal) gravity conditions. Further investigations into the expression levels of angiogenic factors and transcription factors within the CD34+ cell population will elucidate the mechanisms through which temporary microgravity stimulation results in increased EPCs and improves angiogenic potential. Based on the results of this study, the lower-limb ischemia and ulcer model mice can be used to further investigate the effects of microgravity on the angiogenic and tissue-regeneration potentials of QQMNCs. The signal communication systems that are involved in the QQMNCs changes that result from microgravity stimulation could also be investigated. These studies may facilitate the development of better cell cultivation methods.

## Conclusion

Our results suggest that microgravity stimulation can increase the number of CD34+ cells in QQMNCs that are stimulated with microgravity, compared to those that are cultured in an earth (normal) environment. Furthermore, initial cultivation under a microgravity environment followed by cultivation under an earth (normal) environment was shown to remarkably improve EPC expansion rates and angiogenic potential.

PBMNCs that are cultured using QQc combined with exposure to MG and EG environments may be more effective than those cultured with conventional methods. This technique may provide a valuable tool for therapeutic vasculogenesis and tissue regeneration. Indeed, using microgravity conditions may contribute to developments in regenerative medicine using immature stems cells.

## Methods

### Ethical approval

This study was approved by the Clinical Investigation Committee at the Juntendo University School of Medicine, Tokyo, Japan (Ethical Approval number: 17–205). Experiments using human samples were carried out according to all relevant guidelines and regulations of the Juntendo University School of Medicine, Tokyo, Japan. All subjects gave written informed consent prior to participating in this study.

### Subjects

Seven healthy subjects between 20 and 70 years of age were recruited to participate in this study. Subjects with diabetes, a smoking history of >20 cigarettes per day, a collagen tissue malignancy, interventional treatment for coronary or cerebral artery stenosis within six months prior to the study, a haematological disorder, or onset of myocardial infarction or cerebral infarction within six months prior to the study were not included in this study.

### PBMNC isolation and QQc conditions

Peripheral blood (50 mL per subject) was drawn by heparinized venipuncture at the forearm. PBMNCs were isolated by density gradient centrifugation using Histopaque-1077 (Sigma-Aldrich, St. Louis, MO, USA), as previously described^6^.

Cells were cultured using the QQc method, as previously described^6,14^. Briefly, isolated PBMNCs were cultured for seven days at 37 °C in 5% CO_2_ in a six-well Primaria tissue culture plate (BD Falcon; BD Biosciences, San Jose, CA) at a cell density of 2 × 10^6^ cells/2 mL QQc medium per well. QQc medium consisted of Stem Line^®^II (Sigma-Aldrich, St. Louis, MO) media supplemented with the following five human recombinant proteins: stem cell factor (100 ng/mL), thrombopoietin (20 ng/mL), Flt-3 ligand (100 ng/mL), vascular endothelial growth factor (50 ng/mL), and interleukin-6 (20 ng/mL) (all from Peprotec, Rock Hill, NJ, USA).

### 3D-Clinostat and QQc conditions

Microgravity conditions can be simulated on earth by a process called clinorotation, which involves rotating about an axis at a slow rate such that particle freefall is prevented, and the particles are always floating. A 3D–Clinostat (Advanced Engineering Services Co., Ltd., Ibaraki, Japan), which is a multidirectional G force generator, was used for clinorotation. As described by Yuge^18^, the controlled simultaneous rotation of the two axes in the clinostat cancels the cumulative gravity vector at the centre around this piece of equipment, resulting in an environment like that inside a space shuttle. The rotation of a chamber at the centre of the clinostat uniformly disperses the gravity vector within a spherical volume, at a constant angular velocity. Regular tissue culture plates cannot be used in a clinostat, instead disposable cultivation chambers (DCCs; Japan Aerospace Exploration Agency, JAXA, Japan), which are encapsulated cultivation containers, were used.

Cells (5 × 10^6^ cells/5 mL QQ culture medium) were placed in DCCs and rotated at the centre of the 3D-clinostat for seven days at 37 °C in a 5% CO_2_ chamber (Supplementary Fig. S1). Clinostat rotation speed was controlled by a computer at 1.1 rpm (X = outer frame) and 1.3 rpm (Y = inner frame). These speeds were based on previous studies^33^, to cancel gravitational forces. However, the speeds were decreased ten-fold to prevent high agitation in the DCC.

Cells were cultured under the following four different conditions (Fig. 1): NC – normal control (5 × 10^6^ cells/5 mL QQ culture medium in a regular tissue culture plate); EG – earth gravity (5 × 10^6^ cells/5 mL QQ culture medium in a DCC); MG – microgravity (5 × 10^6^ cells/5 mL QQ culture medium in a DCC); and ME – microgravity and earth gravity (5 × 10^6^ cells/5 mL QQ culture medium in a DCC). To simulate ME conditions, cells in DCCs were cultured under microgravity for three days and followed by four days under earth gravity. These experiments were repeated three separate times. After seven days, the cells were collected and their functionality was evaluated.

### Cell expansion

After seven days of culture in QQc medium, cells were collected and live cells counted using trypan blue staining in a haemocytometer under a light microscope. Photographs were also taken of the samples.

### Flow cytometry

Expression of cell surface antigens of the PBMNCs and cells cultured using the QQc method were determined using a flow cytometer (BD FACS Aria™ III Cell Sorter, BD Biosciences, Japan), and the data were analysed with FlowJo software (Tomy Digital Biology Co., Ltd., Tokyo, Japan).

The antibodies and isotype controls used for flow cytometry are listed online in Supplementary Table S2. Cells (5 × 10^5^ cells/100 μL) were suspended in 2 mmol/L of EDTA/2% FBS/PBS buffer and incubated after the addition of 10 μL of FcR blocking reagent at 4 °C for 30 minutes. They were then equally dispensed into reaction tubes for subsequent staining (150 μL/tube). Each aliquot was incubated with 2–3 μL of antibody or isotype control at 4 °C for 30 minutes, and then washed twice with 1 mL of 2 mmol/L of EDTA/2% FBS/PBS buffer. Cells were suspended in 2 mmol/L of EDTA/2% BSA/PBS buffer. Supplementary Fig. S2 shows histograms of isotype and surface staining of CD34+, CD206+ and CCR2+ cells.

### EPC colony-forming assay

The angiogenic potential of PBMNCs and QQMNCs was assessed using the EPC-CFA, as previously described^6,14^. The EPC-CFA was designed to differentiate total EPC colony-forming units (tEPC-CFUs) into two types of EPC-CFUs (primitive and definitive). Primitive EPC-CFU (pEPC-CFUs) are a predominantly proliferative population of cells, and definitive EPCs-CFU (dEPC-CFU) are a predominantly vasculogenic population with greater adhesion, migration, differentiation, and tubularization potential.

Briefly, adhesive EPC colonies were counted using the EPC-CFA (MethoCult SFBIT; Stem Cell Technologies Inc., Vancouver, BC, Canada) with semisolid culture medium containing proangiogenic growth factors/cytokines in 35-mm Primaria dishes (BD Falcon; BD Biosciences). Twelve aliquots of cells were seeded at 2 × 10^5^ cells/dish (three dishes per subject) for EPC-CFA. Sixteen to 18 days after culture initiation, the number of adherent colonies per dish was measured using a gridded scoring dish (Stem Cell Technologies) under phase-contrast light microscopy (Eclipse TE300; Nikon, Tokyo, Japan). The experiments were performed in triplicate. Experimental conditions were masked and two investigators counted the number of pEPC-CFUs and dEPC-CFUs.

### EPC culture assay

PBMNCs and QQMNCs were cultured for seven days in 5% FBS-EGM^®^-2 BulletKit depleted hydrocortisone on human fibronectin-coated Primaria dishes. PBMNCs or QQMNCs were seeded in 96-well plates at 5 × 10^4^ cells in 0.2 mL of EGM-2 medium per well.

Early EPCs were cultured as previously described. Briefly, cells were cultured for seven days. To detect the uptake of 1,1′-dioctadecyl-3,3,3′,3′-tetramethylindocarbocyanine–labelled acetylated LDL (DiI-ac-LDL Biomedical Technologies Inc.), cells were incubated with DiI-ac-LDL (6 μg/mL, 37 °C, 2 h). Cells were then fixed with 4% paraformaldehyde for 10 minutes and incubated with fluorescein-labelled Ulex Europaeus Agglutinin I lectin (Vector Laboratories, Burlingame, CA, USA) for six hours. After staining, cells were examined with an inverted fluorescence microscope (BZ-9000, Keyence, Osaka, Japan), and DiI-ac-LDL and BS-1 lectin double-stained cells (early EPCs) were counted in four random low-power fields.

### Quantitative real-time PCR

Total RNA was isolated from PBMNCs or QQMNCs using the RNeasy Mini kit (Qiagen, Chatsworth, CA). cDNA synthesis was performed with the SensiScript Reverse Transcription kit (Qiagen) as per the manufacturer’s instructions.

The Step One Plus system (Applied Biosystems, Foster City, CA), was used to perform quantitative real-time polymerase chain reactions (qRT-PCR). Briefly, 1 ng/μL diluted cDNA, PowerUp™ SYBR™ green reagents (Applied Biosystem), and 0.5 μmol/L of forward and reverse primers were used for cDNA amplification, as per the manufacturer’s protocol. The relative mRNA expression was calculated using the comparative C_T_ (ΔΔC_T_) method. Measurements were normalized to expression of human *β-actin*. *β-actin* levels themselves did not change between the different groups (Supplementary Fig. S3).

### Statistical analyses

All data are presented as the mean ± SD. The Student’s t-test was used for comparisons between each group. A p-value < 0.05 was considered statistically significant. Prism 6 software (GraphPad Software Inc., Cary, NC) was used to analyse all the data.

## Electronic supplementary material

Supplementary Information

## Data Availability

All data generated or analysed during this study are included in this published article (and its Supplementary Information files).
