# Supplementary material for: Effectiveness of endothelial progenitor cell culture under microgravity for improved angiogenic potential
Source: Sci Rep. 2018 Sep 24;8:14239. doi: 10.1038/s41598-018-32073-2 (PMC6155294; doi:10.1038/s41598-018-32073-2)
Supplement: Supplementary file 1 — Supplementary Information [file 41598_2018_32073_MOESM1_ESM.pdf]

Supplementary Information

Effectiveness of endothelial progenitor cell culture under microgravity for improved angiogenic potential

Hiroko Hagiwara, Akira Higashibata, Shiho Ogawa, Shigeyuki Kanazawa, Hiroshi Mizuno & Rica Tanaka

Supplementary Table S1. Expression levels of surface markers by flow cytometry analysis of MNC and QQMNC

%	PBMNC	NC	EG	MG	ME
CD34	0.27 ± 0.29	1.12 ± 0.61	1.38 ± 3.02	4.90 ± 2.71*†	5.50 ± 3.68*†
CD206	4.12 ± 7.28	28.62 ± 1.61	12.02 ± 3.30**	12.11 ± 4.08**	9.35 ± 4.58**
CCR2	21.28 ± 9.54***	0.98 ± 0.83	0.30 ± 0.39	0.20 ± 0.27	0.19 ± 0.29
CXCR4	75.0 ± 21.21	89.80 ± 2.79	89.73 ± 2.35	91.20 ± 2.78	88.7 ± 0.76
CD31	48.53 ± 18.90	68.40 ± 5.07	56.03 ± 6.62	57.14 ± 10.22	56.92 ± 8.04*
CD3	56.02 ± 7.49	53.88 ± 21.49	51.39 ± 30.60	68.75 ± 8.61	65.69 ± 5.36

*:p < 0.05 *vs*. NC, **:p < 0.01 *vs*. NC, ***:p < 0.005 *vs*. NC,

†:p < 0.05 *vs*. EG

Supplementary Table S2. Antibodies (A) and isotype (B) antibodies used in flow cytometry

A

Antibody	Isotype	Company, Catalog No.
CD34-PE	Mouse IgG1	BioLegend, 343506
CD206-PE-Cy7	Mouse IgG1	BioLegend, 400125
CD31-FITC	Mouse IgG1	BioLegend, 303104
CXCR4-APC	Mouse IgG1	BD, 555976
CD3-Alexa700	Mouse IgG1	BioLegend, 300424
CD4-FITC	Mouse IgG1	Biolegend, 300506
CD8a-Brilliant violet421	Mouse IgG1	BioLegend, 301036
CD14-APC-Cy7	Mouse IgG1	BioLegend, 325620
CCR2-PerCP-Cy5.5	Mouse IgG2a	BioLegend, 357204
CD133-APC	Mouse IgG1	Miltenyi Biotec, 130-090-826

PE = phycoerythrin; APC = allophycocyanin; CXCR4 = C-X-C chemokine receptor type 4; CCR2 = chemokine receptor 2

B

Antibody	Company, Catalog No.
Mouse IgG1 κ-PE	BD, 555749
Mouse IgG1 κ-PE-Cy7	BioLegend, 400252

Mouse IgG1 κ-FITC	BD, 555748
Mouse IgG1 κ-APC	Beckman-Coulter, IM2475
Mouse IgG1 κ-Alexa700	BioLegend, 400144
Mouse IgG1 κ-Brilliant violet421	BioLegend, 400158
Mouse IgG1 κ-APC-Cy7	BioLegend, 400128
Mouse IgG2a κ-PerCP-Cy5.5	BioLegend, 357204

PE = phycoerythrin; APC = allophycocyanin

Supplementary Table S3. Human PCR primers for qRT-PCR in MNC and QQMNC

Gene	Forward Primer	Reverse Primer
VEGF-A	5’-TCACCATGCAGATTATGCGGA -3’	5’-ACCAACGTACACGCTCCAG -3’
VEGF-B	5’-AGGTGACACATGGCTTTTCAG -3’	5’-GTTCCCCCACTGGGATATAGC-3’
VEGF-R2 / KDR	5’-GGCCCAATAATCAGAGTGGCA-3’	5’-CCAGTGTCATTTCCGATCACTTT-3’
VEGF-R1 / Flt1	5’-TTTGCCCGAAATGGTGAGTAAGG-3’	5’-TGGTTTGCTTGAGCTGTGTTC-3’
ANG-1	5’-GCCCTAAGCCATCAGCAATC -3’	5’-GGTTGCACATCCAAGCCAAG-3’
ANG-2	5’-ACCTGTTGAACCAAACAGCG-3’	5’-GTCGAGAGGGAGTGTTCCAAG-3’
IGF	5’-GCCCAAAATGCACTGATGTAAA-3’	5’-AGTGACTTTGCTATGAGTTGGTGAGT-3’
HGF	5’-CGTAGCGTACCTCTGGATTGC-3’	5’-GCTATCGGGGTAAAGACCTACA-3’
PDGF	5’-GCAAGACCAGGACGGTCATTT-3’	5’-GGCACTTGACACTGCTCGT-3’
FGF-1	5’-AATGGGAGCTGCAAACGCGGTCC-3’	5’-TCAACCAGGTGAGGACCCCTCGA-3’

MMP2	5’-GGTTCCCCTGTTCACTCTACTTAGC-3’	5’-CGGCTTGGTTTTCCTCCAT-3’
MMP9	5’-AGACCTGGGCAGATTCCAAAC-3’	5’-CGGCAAGTCTTCCGAGTAGT-3’
SOD	5’-TTTGCGTCGTAGTCTCCTGC -3’	5’-CCACACCTTCACTGGTCCAT-3’
e-NOS	5’-TACAGGCTAAAACCTTAGAAGAGGA-3’	5’-CTGACAGCTTCCAGATGCC-3’
TNF-α	5’-GAGACCAGGGAGCCTTTGGT-3’	5’-TGTGTCAATTTCTAGGTGAGGTCTTC-3’
Leptin	5’-TCACTAGATGGCGAGCATCCT -3’	5’-CACGCTCAGCTAACTTTTGTGTTT-3’
β-actin	5’-GTCATTCCAAATATGAGATGCGTTG-3’	5’-TGTGGACTTGGGAGAGGACT-3’

qRT-PCR = quantitative real-time polymerase chain reaction; ANG = angiopoietin; IGF = insulin-like growth factor; HGF = hepatocyte growth factor; PDGF = platelet-derived growth factor; FGF = fibroblast growth factor; MMP = matrix metalloprotease; SOD = super oxide dismutase; NOS = nitric oxide synthase; TNF = tumour necrosis factor.

Supplementary Figure S1:

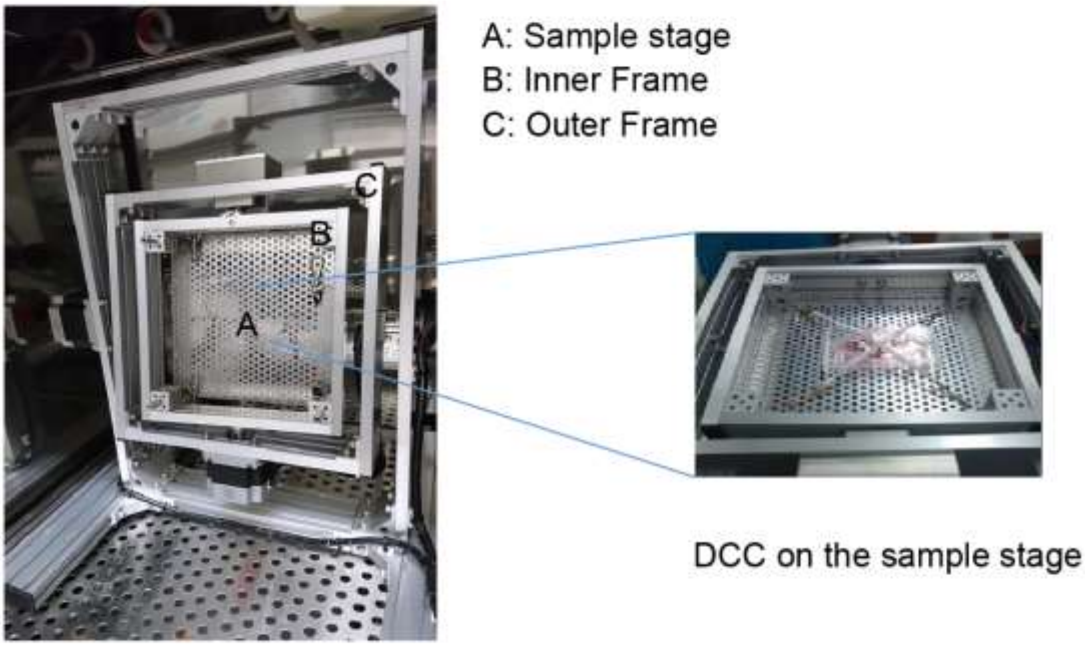

Fig S1: Photograph of the 3D-clinostat. The image shows the inner and outer frames and sample stage with a disposable cultivation chamber (DCC).

Supplementary Figure S2:

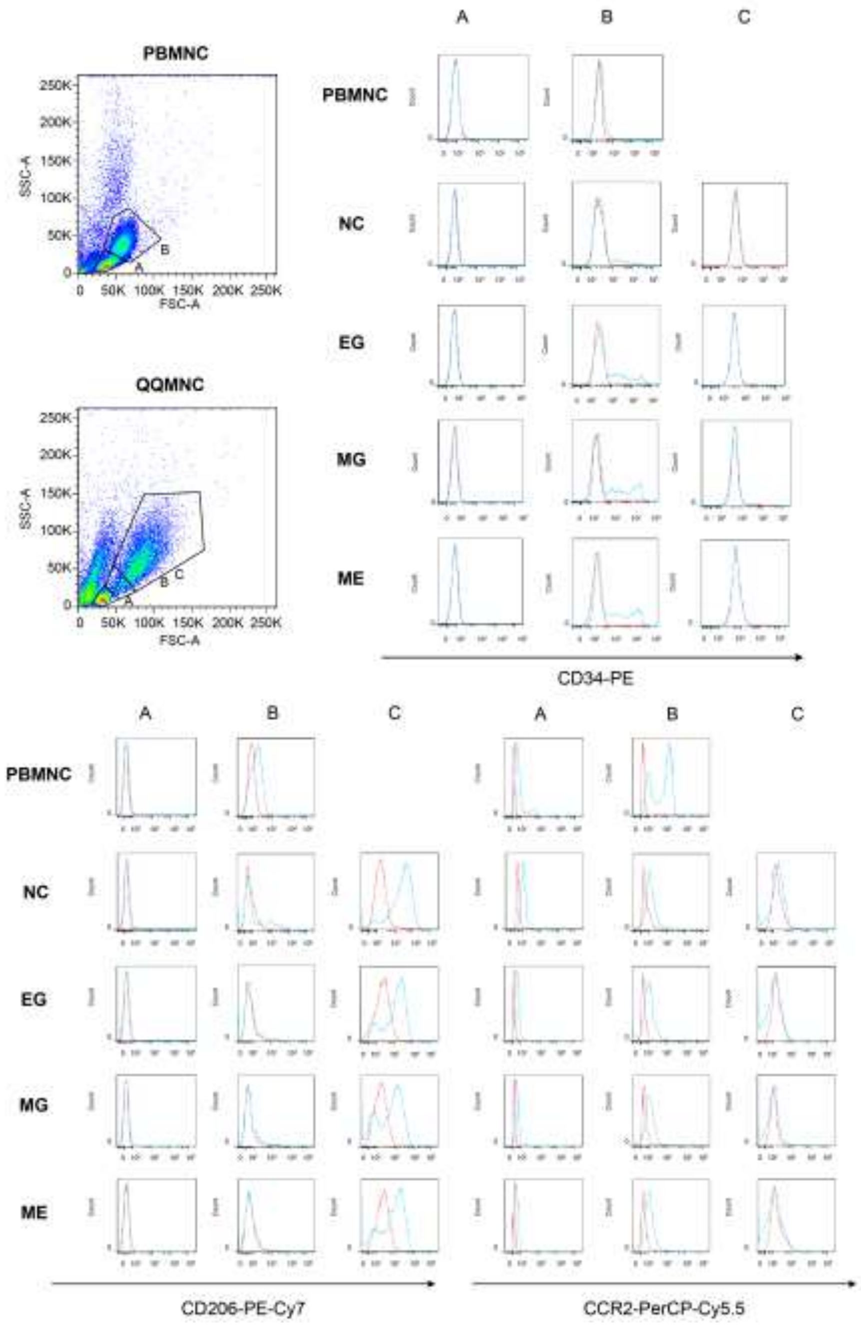

Fig S2: Flow cytometry data for surface staining of CD34, CD206, and CCR2. After culturing for 7 days, PBMNC showed 2 cell populations whereas there were 3 populations for QQMNC. We first gated the cells into these populations and labelled them as A, B, and C. Positive cells were determined by histogram overlays using isotype controls as a sum of the percentage of cells in gates A, B and C for QQMNC or the percentage of cells in gate A and B for PBMNC.

Supplementary Figure S3:

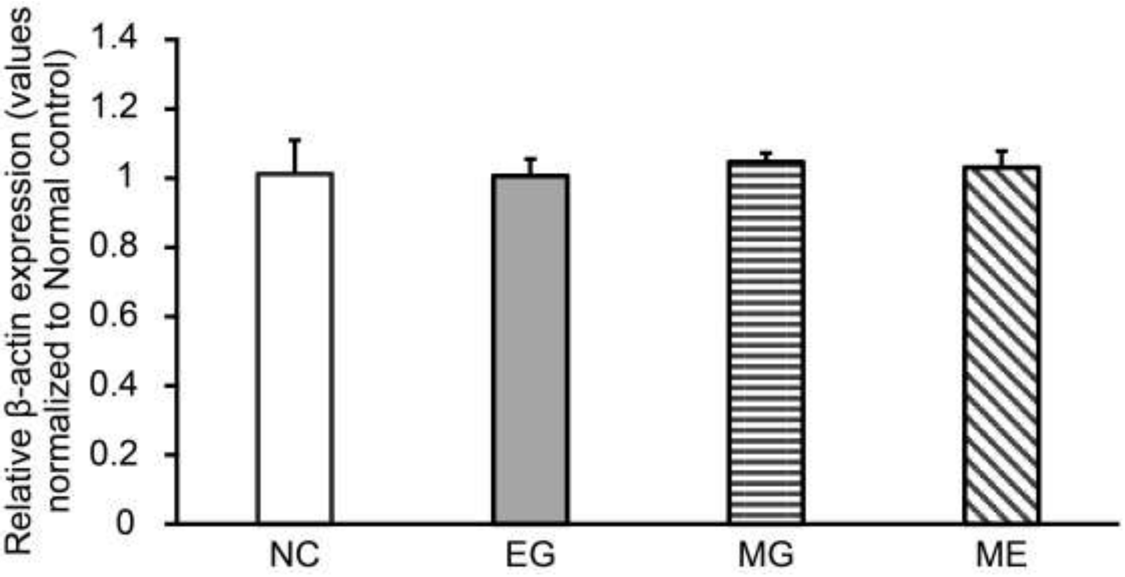

Fig. S3: Quantitative PCR for QQMNC under different gravity conditions for β-actin. Values represent mean ± SD from 21 culture samples.
